# Entropic Analysis of Mirror Symmetry Breaking in Chiral Hypercycles

**DOI:** 10.3390/life9010028

**Published:** 2019-03-15

**Authors:** David Hochberg, Josep M. Ribó

**Affiliations:** 1 Centro de Astrobiología (CSIC-INTA), Department of Molecular Evolution, Carretera Ajalvir Kilómetro 4, 28850 Torrejón de Ardoz, Madrid, Spain; 2 Department of Organic and Inorganic Chemistry, Institute of Cosmos Science (IEEC-UB), University of Barcelona, 08028 Barcelona, Catalonia, Spain; jmribo@ub.edu

**Keywords:** spontaneous mirror symmetry breaking, chiral replicators, entropy production, general evolution criterion, non-equilibrium thermodynamics, stoichiometric network analysis

## Abstract

Replicators are fundamental to the origin of life and evolvability. Biology exhibits homochirality: only one of two enantiomers is used in proteins and nucleic acids. Thermodynamic studies of chemical replicators able to lead to homochirality shed valuable light on the origin of homochirality and life in conformity with the underlying mechanisms and constraints. In line with this framework, enantioselective hypercyclic replicators may lead to spontaneous mirror symmetry breaking (SMSB) without the need for additional heterochiral inhibition reactions, which can be an obstacle for the emergence of evolutionary selection properties. We analyze the entropy production of a two-replicator system subject to homochiral cross-catalysis which can undergo SMSB in an open-flow reactor. The entropy exchange with the environment is provided by the input and output matter flows, and is essential for balancing the entropy production at the non-equilibrium stationary states. The partial entropy contributions, associated with the individual elementary flux modes, as defined by stoichiometric network analysis (SNA), describe how the system’s internal currents evolve, maintaining the balance between entropy production and exchange, while *minimizing* the entropy production after the symmetry breaking transition. We validate the General Evolution Criterion, stating that the change in the chemical affinities proceeds in a way as to *lower* the value of the entropy production.

## 1. Introduction

The importance of studying chemical systems subject to various architectures, and capable of spontaneous mirror symmetry breaking (SMSB), owes to the problem of the origin of biological homochirality, an outstanding problem in origin of life research [1,2], as well as a crucial factor to take into account in the design of synthetic systems mimicking primordial processes of life. The current consensus is that the homochirality of biological compounds: the observed bias in biopolymers made up from homochiral L-amino acids and D-sugars is a condition associated with life that most likely emerged through processes of spontaneous mirror symmetry breaking very early on during abiotic chemical evolution [2]. Homochirality is ubiquitous and present in biological chemistry right from the outset.

Spontaneous mirror symmetry breaking, or absolute asymmetric synthesis (AAS), refer to the transformation of achiral or racemizing initial products to final chiral reaction products in detectable enantiomeric excesses, and in the absence of any chiral polarization or external chiral forces and influences. SMSB and AAS involve transformations yielding nonracemic (scalemic) outcomes as non-equilibrium steady states (NESS) [3,4,5,6]. This phenomenon may occur in enantioselective-autocatalytic reaction networks leading to a bifurcation scenario [7,8]. Within the framework of non-equilibrium non-linear thermodynamics of irreversible processes [9,10], mirror symmetry breaking can occur for specific system parameters and only when the system is kept out of thermodynamic equilibrium with its surroundings [3]. The racemic state becomes metastable along the thermodynamic branch and inevitable chiral fluctuations perturb the system to make a transition to one of two energetically degenerate chiral states: a bifurcation to ordered states takes place with a consequent decrease in the symmetry and for which the production of entropy is minimized.

Reaction networks able to lead to SMSB require, as a necessary but not sufficient condition, nonlinear kinetic dependences of enantioselective autocatalysis. Although a rare reaction in laboratory chemistry [11,12], it is significant in life, because it sustains self-reproduction (i.e., via replicators) in the nucleic acids/protein domain. Indeed, replicators are believed to be fundamental to the origin of life [13], whose survival hinges on the accuracy of their replication and growth efficiency. Autocatalysis is a basic property of life [14,15,16]. Thus the emergence of autocatalysis during the formation of the first replicators represents a crucial stage in chemical evolution. Biological replication is enantioselective, therefore an SMSB scenario of enantioselective autocatalysis occurring at the same stage of abiotic evolution, rather than at the stage of the emergence of replicators (pre-RNA- or RNA-world), is a reasonable hypothesis which has been taken up and developed in [17]. The quest to understand the origin of biological homochirality in replicator dynamics makes the study of such chemical networks especially interesting and relevant.

Enantioselective hypercycles enable quadratic (first order) autocatalysis to achieve the enantioselective behavior of cubic (second-order) autocatalysis, and therefore may lead to spontaneous mirror symmetry breaking (thus resulting in a chiral final stationary state instead of a racemic one) for specific reaction rate constants in systems with thermodynamic architectures that maintain them out of equilibrium with their surroundings [17]. The significance of such a SMSB reaction network is that it does not imply heterochiral inhibiting reactions, such as those of the Frank-like models, and as a consequence, the emergence of biological homochirality could already be included, both theoretically and experimentally, in the current models of the selection and evolution of biological replicators. These results [17] suggest an abiotic scenario of a *simultaneous* emergence of biological homochirality during the formation of replicator networks with catalytic activity (such, as for example, in the RNA-world). Furthermore, such an hypothesis also implies that the current chiral machinery present in extant living beings, capable of transferring chirality and which is highly resistant to racemization, is a complex SMSB network which evolved from the primordial ones.

The question of biological homochirality requires an understanding of the non-equilibrium thermodynamic conditions that may lead to stable deviations from the racemic composition. The purpose of this work is to place a strong emphasis on the entropic changes and exchanges associated with SMSB. In so doing we will understand how such chemical dynamical systems can be conceived and in strict obeyance of the thermodynamic laws. This approach is developed in detail employing a model of two cross-catalyzed chiral replicators. This may be regarded as a primitive infrabiological system [13] built out of two coupled (auto)catalytic systems. This will lead to a description of entropy production, entropy exchange, and the *balance* of the former and the latter at a NESS, for non-equilibrium systems in terms of the extreme flux modes, as defined by stoichiometric network analysis (SNA) [18].

## 2. Enantioselective Replicators in Open-Flow Reactors

The so-called hypercycle model [19] solves the problem of how to perform replicator selection, that is, how to achieve exponential growth dynamics based on quadratic autocatalysis. Recently, we have shown that the model is also able to yield spontaneous mirror symmetry breaking (SMSB) in a number of hypercyclic networks of varying complexity, and this means enantiomeric selection in the case of chiral replicators [17]. The simplest reaction network of hypercyclic enantioselective autocatalysis is that involving two chiral replicators ${}^{1}R,{}^{2}R$, and is the model we will thoroughly investigate here:

(1) $$\begin{array}{cc} A+{}^{1}R_{D}+{}^{2}R_{D}\overset{k_{1}}{\underset{k_{2}}{⇌}}2\;\;{}^{1}R_{D}+{}^{2}R_{D} & \;A+{}^{1}R_{L}+{}^{2}R_{L}\overset{k_{7}}{\underset{k_{8}}{⇌}}2\;\;{}^{1}R_{L}+{}^{2}R_{L} \end{array}$$

(2) $$\begin{array}{cc} A+{}^{2}R_{D}+{}^{1}R_{D}\overset{k_{3}}{\underset{k_{4}}{⇌}}2\;\;{}^{2}R_{D}+{}^{1}R_{D} & \;A+{}^{2}R_{L}+{}^{1}R_{L}\overset{k_{9}}{\underset{k_{10}}{⇌}}2\;\;{}^{2}R_{L}+{}^{1}R_{L} \end{array}$$

(3) $$\begin{array}{cc} {}^{1}R_{D}\overset{k_{f}\left(5\right)}{⟶}\emptyset & \;{}^{1}R_{L}\overset{k_{f}\left(11\right)}{⟶}\emptyset \end{array}$$

(4) $$\begin{array}{cc} {}^{2}R_{D}\overset{k_{f}\left(6\right)}{⟶}\emptyset & \;{}^{2}R_{L}\overset{k_{f}\left(12\right)}{⟶}\emptyset \end{array}$$

(5) $$\begin{array}{ccc} \bar{\emptyset } & \overset{k_{f}{\left[A\right]}_{0}\left(13\right)}{⟶} & A \end{array}$$

(6) $$\begin{array}{ccc} A & \overset{k_{f}\left(14\right)}{⟶} & \emptyset . \end{array}$$

**Figure 1:**
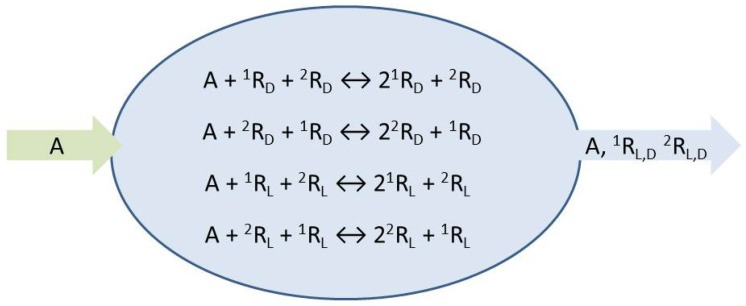
Reaction network for two cross-catalyzed chiral replicators in an open flow reactor.

The species *A* represents the achiral resources needed to form the chiral replicators which engage in homochiral mutual cross-catalysis, Equations (1) and (2). We place these micro-reversible reactions within an open flow system, Figure 1, which implies the entry of a specific volume of reactant solution and the exit of the same volume of the actual concentrations (those determined dynamically within the continuously stirred tank reactor or CSTR) of all the reaction network species. The symbols $\bar{\emptyset },\emptyset$ represent environments external to the reactor from which chemical species can be input, or flow to, respectively: see Equations (3)–(6). The reaction rate constants are determined below.

The resultant fourteen transformations are numbered sequentially as indicated, a convenience for setting up the stoichiometric network analysis (SNA) [18] that follows. SNA is a systematic and powerful general approach for treating nonlinear dynamics of chemical reaction mechanisms based on network stoichiometry. We obtain the stoichiometric matrix ν: the transformations, including both the reactions and the flows, are represented by the columns, numbered from 1 to 14, whereas the rows, from top to bottom, represent the five species in this order ${\left\{X_{i}\right\}}_{i=1}^{5}=\left\{A,{}^{2}R_{D},{}^{1}R_{D},{}^{1}R_{L},{}^{2}R_{L}\right\}$:

(7) $$\nu =\left(\begin{array}{cccccccccccccc} -1 & 1 & -1 & 1 & 0 & 0 & -1 & 1 & -1 & 1 & 0 & 0 & 1 & -1\\ 0 & 0 & 1 & -1 & 0 & -1 & 0 & 0 & 0 & 0 & 0 & 0 & 0 & 0\\ 1 & -1 & 0 & 0 & -1 & 0 & 0 & 0 & 0 & 0 & 0 & 0 & 0 & 0\\ 0 & 0 & 0 & 0 & 0 & 0 & 1 & -1 & 0 & 0 & -1 & 0 & 0 & 0\\ 0 & 0 & 0 & 0 & 0 & 0 & 0 & 0 & 1 & -1 & 0 & -1 & 0 & 0 \end{array}\right).$$

The chemical pathway structure of the reaction plus flow scheme Equations (1)–(6) is provided from knowledge of the right null space of ν (and when this null space is *intersected* with the positive orthant of a 14-dimensional Euclidean space), and corresponds to the set of all the stationary state reaction rates [18]. Knowledge of the stationary, or steady, states reveals the explicit reaction pathways and the flow patterns and matter currents of the chemical network. This is therefore an ideal tool to use for exploring SMSB. Carrying out these preliminary algebraic operations (the extreme flux modes, EFMs, are determined from ν using the freely available program package COPASI [20]) yields a set of nine vectors, the extreme flux modes (EFM) ${\left\{E_{i}\right\}}_{i=1}^{9}$, which can be organized as the columns of the following matrix E:

(8) $$E=\left(\begin{array}{ccccccccc} 0 & 0 & 1 & 0 & 0 & 0 & 0 & 1 & 0\\ 0 & 0 & 1 & 0 & 0 & 0 & 0 & 0 & 0\\ 0 & 0 & 0 & 1 & 0 & 0 & 0 & 0 & 1\\ 0 & 0 & 0 & 1 & 0 & 0 & 0 & 0 & 0\\ 0 & 0 & 0 & 0 & 0 & 0 & 0 & 1 & 0\\ 0 & 0 & 0 & 0 & 0 & 0 & 0 & 0 & 1\\ 0 & 1 & 0 & 0 & 0 & 0 & 1 & 0 & 0\\ 0 & 1 & 0 & 0 & 0 & 0 & 0 & 0 & 0\\ 1 & 0 & 0 & 0 & 0 & 1 & 0 & 0 & 0\\ 1 & 0 & 0 & 0 & 0 & 0 & 0 & 0 & 0\\ 0 & 0 & 0 & 0 & 0 & 0 & 1 & 0 & 0\\ 0 & 0 & 0 & 0 & 0 & 1 & 0 & 0 & 0\\ 0 & 0 & 0 & 0 & 1 & 1 & 1 & 1 & 1\\ 0 & 0 & 0 & 0 & 1 & 0 & 0 & 0 & 0 \end{array}\right),$$

and which satisfies νE=0. These nine EFM vectors and the reaction pathways they represent are listed in Table 1. The first four flux modes, $E_{1}$ to $E_{4}$, correspond to the internal fluxes describing the forward and reverse catalytic activity of each one of the enantiomers of the two replicators. The flux mode $E_{5}$ corresponds to the flux of the achiral species A traversing the reactor in the absence of any reaction. The final four flux modes $E_{6}$ to $E_{9}$ correspond to the unidirectional transformation fluxes of A to one of the enantiomers of the two replicators ${}^{1}R,{}^{2}R$, followed by the outflow from the reactor of the given enantiomer.

The reaction rate monomial corresponding to the *j*-th transformation (j=1,2,…,14) in Equations (1)–(6) can be written as follows, where $\alpha_{ji}$ is the stoichiometric coefficient of the *i*th species in the *j*th transformation:

(9) $$v_{j}\left(k_{j},x\right)=k_{j}\prod_{i=1}^{5}{\left[X_{i}\right]}^{\alpha_{ji}}.$$

Then, from SNA, the most general vector of the *stationary state* reaction rates $v_{ss}$, whose components are given by Equation (9) for the stationary concentrations ${\left[X_{i}\right]}_{ss}$, can be expressed as a linear combination of the EFM vectors with nonnegative expansion coefficients $j_{i}\geq 0$ [18] as follows:

(10) $$v_{ss}=\sum_{i=1}^{9}j_{i}\;E_{i}=\left(j_{3}+j_{8},j_{3},j_{4}+j_{9},j_{4},j_{8},j_{9},j_{2}+j_{7},j_{2},j_{1}+j_{6},j_{1},j_{7},j_{6},j_{5}+j_{6}+j_{7}+j_{8}+j_{9},j_{5}\right).$$

Each non-negative parameter $j_{i}>0$ represents the magnitude of the current flowing through the *i*th flux mode, or pathway: $E_{i}$. The virtue of this SNA representation is that all steady state properties of the full network can be expressed as functions of these flux magnitudes.

A SNA stability analysis of the racemic configuration can be carried out following the procedure established in [21], and leads to conditions in terms of these flux magnitude parameters $j_{i}$. The instability of the racemic state is driven by the initially racemic open flow currents $j_{6}=j_{9}>0$ and $j_{7}=j_{8}>0$. These parameters multiply the EFMs $E_{6}$ to $E_{9}$, respectively, which from Table 1, involve the *unidirectional* sequences of transformations that drive the net production of the enantiomers via the forward mutual cross-catalyses. These initially racemic currents drive the instability of the racemic configuration. These four currents result from (i) the inflow of achiral resource A, (ii) its conversion into enantiomers of the replicators, and followed by (iii) the outflow of these enantiomers from the reactor. After SMSB, these racemic flux equalities are broken, indicating that relatively more flux flows through one of the corresponding flux mode pathway than through its oppositely handed flux mode pathway. This can be appreciated clearly, in for example, the SMSB bifurcations revealed by the partial entropy productions for each EFM, see Figures 4 and 5. After SMSB, we have $j_{1}>j_{4}$, $j_{2}>j_{3}$ and $j_{6}>j_{9}$, $j_{7}>j_{8}$: hence all the L-handed fluxes (and hence their partial entropy productions) are greater than their D-handed counterparts; see Table 1 for the EFMs involved.

By marked contrast, if only the achiral species *A* is allowed to enter and exit the reactor, but none of the replicators, then SNA proves that the racemic state is absolutely stable, and for all values of $j_{1}=j_{4}>0,j_{2}=j_{3}>0,j_{5}>0$. This latter situation corresponds to preventing the enantiomer outflow Equations (3) and (4) from the reactor; the corresponding stoichiometric matrix then gives rise to only the first five EFMs listed in Table 1. The latter four EFMs are simply not present. The first four of which all correspond to *closed* “unproductive” pathways, while the fifth is the unreactive flow-through of the resource A. In this case, the non-equilibrium racemic states (which define the thermodynamic branch) are the only stable outcomes: the equalities $j_{1}=j_{4}>0,j_{2}=j_{3}>0$ are never broken.

**Figure 2:**
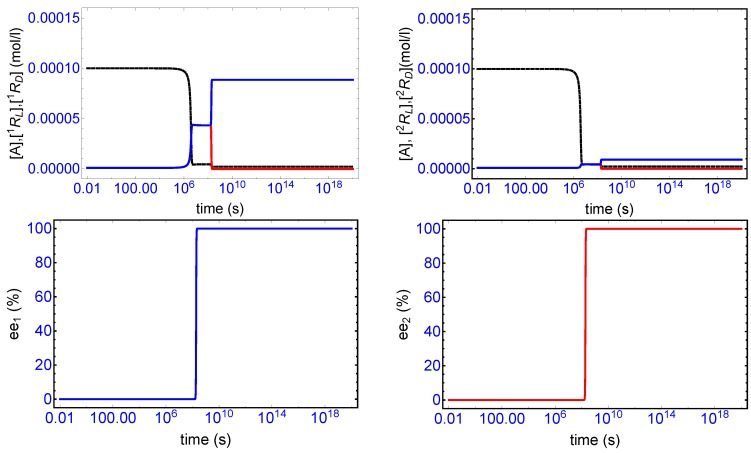
Spontaneous mirror symmetry breaking (SMSB) in a simple two-hypercycle network Equations (1)–(6) in an open-flow reactor of volume V=1 L. The two homochiral cross-catalyzed enantioselective replicators ${}^{1}R$ and ${}^{2}R$ are fed by a common achiral resource A. The reaction rate constants are $k_{a}=1\times 10^{4}$, $k_{-a}=1\times 10$, $k_{b}=1\times 10^{3}$, $k_{-b}=5\times 10^{-1}$, see text. Initial resource concentration in the reactor and in the constant input volume (*f* = 0.2 μL/s): ${\left[A\right]}_{in}=1\times 10^{-4}$ mol/L. Initial replicator concentrations in the reactor ${\left[{}^{1}R_{L}\right]}_{0}={\left[{}^{1}R_{D}\right]}_{0}={\left[{}^{2}R_{L}\right]}_{0}={\left[{}^{2}R_{D}\right]}_{0}=$$1\times 10^{-6}$ mol/L and the initial chiral fluctuation is simulated by an incremental concentration of $\delta {\left[{}^{2}R_{L}\right]}_{0}=1\times 10^{-23}$ mol/L in the ${}^{2}R_{L}$ enantiomer. **Top row**: formation of the enantiomers from the initial input concentration of resource and the symmetry breaking bifurcation. Black dashed curves are [A], blue curves are $\left[R_{L}\right]$, and the red curves are $\left[R_{D}\right]$ for each replicator. The SMSB event occurs at approximately $t\approx 2\times 10^{8}$ s. **Bottom row**: percent enantiomeric excess (ee%) for each replicator reaches 100% homochirality. The rise in ee initiates approximately at $t\approx 2\times 10^{8}$ s. Qualitatively similar behavior is obtained when direct production and autocatalysis of the replicators are included in this scheme, as well as for other hypercyclic networks involving more replicators, and also for other system architectures [17].

The reaction rate constants in Equations (1) and (2) are constrained by chirality: this implies $k_{1}=k_{7}=k_{a},k_{2}=k_{8}=k_{-a}$ and $k_{3}=k_{9}=k_{b},k_{4}=k_{10}=k_{-b}$. Figure 2, top row, displays a characteristic example of SMSB for the two-replicator network in a open flow reactor, and for the rate constants and initial conditions indicated there. The final enantiomeric excesses achieved for each replicator, see bottom row Figure 2, where $ee_{1}\left(\%\right)=\frac{\left[{}^{1}R_{L}\right]-\left[{}^{1}R_{D}\right]}{\left[{}^{1}R_{L}\right]+\left[{}^{1}R_{D}\right]}\times 100\%$ etc., is that of 100% homochirality.

There is a relatively long induction period for which both replicators remain close to their initially extremely low concentration values, followed by a relatively rapid consumption of the shared achiral species A (the dashed black curves, top Figure 2) which increases the racemic concentrations to higher levels for each replicator. This increased level of non-equilibrium racemic composition then becomes unstable leading to a bifurcation in the enantiomeric concentrations. In this example, we have $\left[{}^{1}R_{L}\right]>>\left[{}^{1}R_{D}\right]$ (top left) and $\left[{}^{2}R_{L}\right]>>\left[{}^{2}R_{D}\right]$ (top right) since we subjected the system to an initial minuscule perturbation $\delta {\left[{}^{2}R_{L}\right]}_{0}$ in just one of L-handed enantiomers. The initial minuscule chiral fluctuation ($ee_{0}=10^{-16}\%$) has become amplified to a full 100% homochirality.

## 3. Entropy Production

The entropy production due to *k*-reversible reactions can be expressed in terms of their corresponding forward $v_{f}$ and reverse $v_{r}$ reaction rates, employing Equation (9), as follows [10]:

(11) $$\sigma =R\sum_{k}\left(v_{k,f}-v_{k,r}\right)\ln\left(\frac{v_{k,f}}{v_{k,r}}\right)\geq 0,$$

where *R* is the ideal gas constant. For our replicator scheme, when applied to the four reversible reactions as enumerated in Equations (1) and (2), this leads to

(12) $$\sigma /R=\left(v_{1}-v_{2}\right)\ln\left(\frac{v_{1}}{v_{2}}\right)+\left(v_{3}-v_{4}\right)\ln\left(\frac{v_{3}}{v_{4}}\right)+\left(v_{7}-v_{8}\right)\ln\left(\frac{v_{7}}{v_{8}}\right)+\left(v_{9}-v_{10}\right)\ln\left(\frac{v_{9}}{v_{10}}\right)\geq 0,$$

where the indicated reaction rates $v_{j}$ are given by Equation (9).

SNA reveals the specific reaction pathways that are responsible for producing entropy [22]. Thus from Equation (10), for any nonequilibrium stationary state (NESS), we find that the entropy production depends on the following flux mode magnitudes *j* and in the following way:

(13) $${\sigma /R|}_{NESS}=j_{8}\ln\left(\frac{j_{3}+j_{8}}{j_{3}}\right)+j_{9}\ln\left(\frac{j_{4}+j_{9}}{j_{4}}\right)+j_{7}\ln\left(\frac{j_{2}+j_{7}}{j_{2}}\right)+j_{6}\ln\left(\frac{j_{1}+j_{6}}{j_{1}}\right)\geq 0.$$

Therefore, the entropy production is driven exclusively by the *unidirectional* extreme flux modes $E_{6}$, $E_{7}$, $E_{8}$, and $E_{9}$, see Table 1, and whose flux magnitudes are specified by the nonnegative coefficients $j_{6},j_{7},j_{8}$, and $j_{9}$, respectively. Note each individual term above is positive definite. By contrast, the four internal closed EFMs $E_{1}$, $E_{2}$, $E_{3}$, and $E_{4}$ do contribute to the production of entropy—but *only* and necessarily in the presence of the open unidirectional modes—and cannot produce entropy of and by themselves. Note furthermore that the input/output unreactive flow of resource *A* plays no role whatsoever in the production of entropy, since Equation (13) does not depend on $j_{5}>0$. At least one open *productive* unidirectional pathway must be operative in order that the system produce entropy [23].

**Figure 3:**
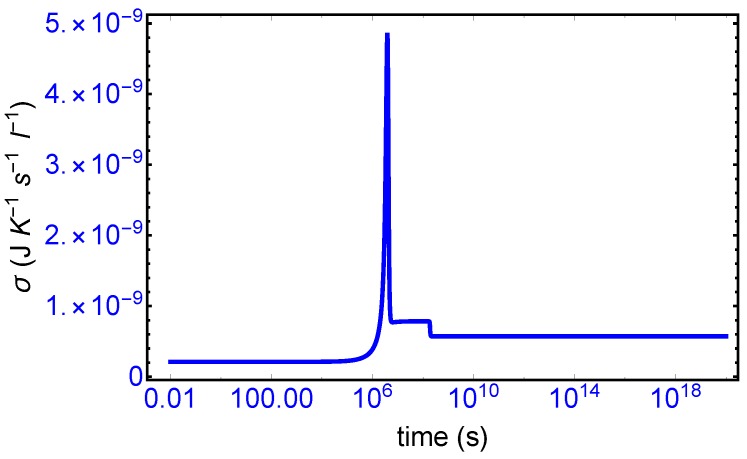
The entropy production Equation (11) over the full time range of the simulation of Figure 2. The first pronounced peak at $t\approx 10^{6}$ s corresponds to the almost complete conversion of A into both the enantiomers of each replicator. The production then decreases and levels off to a narrow plateau corresponding to the unstable racemic state, and then subsequently decreases once more to a final minimum value, with respect to this former unstable plateau, immediately after the symmetry breaking bifurcation occurring at $t\approx 2\times 10^{8}$ s. Compare the time scales here to those in the top row of Figure 2.

The evolution of the entropy production over the full time range of the simulation is given in Figure 3. The various time scales that can be appreciated there can be compared with those marking the evolution of the enantiomers in Figure 2. The production remains relatively low during the initial induction period $0<t<10^{6}$ s, as there is little transformation of resource *A* to the enantiomers. The pronounced production peak near $t\approx 10^{6}$ s corresponds to the rapid conversion of resource to enantiomers via the cross-catalysis, which increases the level of the, as yet, racemic concentrations. Once this conversion process has all but halted, due to the diminished supply of resource, the system then remains in a new meta-stable racemic configuration: this is seen in the narrow entropy production “plateau” in Figure 3 and corresponds to the higher racemic concentration profile located in time range of approximately $10^{7}<t<10^{8}$ s. The mirror symmetry breaking bifurcation in the enantiomeric concentrations (see Figure 2) is accompanied by a drop in the entropy production to its final stationary value in the homochiral state. The entropy production is *minimized* in the chiral state, relative to the entropy production in the prior meta-stable racemic state. Such a qualitative trend has been observed before for other types of reactions [5,10,24,25].

## 4. Entropy Production of the Extreme Flux Modes

We can define and calculate the *partial* entropy productions and exchanges [22] for each individual irreversible transformation, as indicated in Table 2. This reveals how the entropy production and exchange are partitioned along all the pathways of a reaction network. The first two transformations in Table 2 represent the forward and reverse cross-catalysis, with rate constants $k_{+},k_{-}$, respectively. The latter two correspond to a fixed input concentration ${\left[X\right]}_{in}$, and an output flow of [Y], where ${\left[..\right]}_{eq}$ denotes the equilibrium concentration of the indicated species. These are the concentrations the system would relax to if the reactor, containing the chemical mass of the final NESS, were to be isolated from the open flow. These equilibrium concentrations are used to define the relative chemical potential [22]. The flow parameter f=q/V, where *q* is the volume of fluid flowing into and out of the reactor of volume *V*.

All the individual transformations in Equations (1)–(6) belong to one of these four forms listed in Table 2. Then the entropic contributions corresponding to each extreme flux mode vector $E_{i}$ can be defined and calculated [22] using Table 1 and Table 2, and which results in weighted linear combinations of partial entropy productions due to both the chemical reactions and the exchange fluxes. Carrying this out yields the partial entropies corresponding to the individual EFM vectors as follows:

(14)σ(E1)=σ(22RL+1RL→A+2RL+1RL)+12σ(A+2RL+1RL→22RL+1RL)(15)σ(E2)=σ(21RL+2RL→A+1RL+2RL)+12σ(A+1RL+2RL→21RL+2RL)(16)σ(E3)=σ(21RD+2RD→A+1RD+2RD)+12σ(A+1RD+2RD→21RD+2RD)(17)σ(E4)=σ(22RD+1RD→A+2RD+1RD)+12σ(A+2RD+1RD→22RD+1RD)(18)σ(E5)=σ(A→∅)+15σ(∅→A)(19)σ(E6)=15σ(∅¯→A)+σ(2RL→∅)+12σ(A+2RL+1RL→22RL+1RL)(20)σ(E7)=15σ(∅¯→A)+σ(1RL→∅)+12σ(A+1RL+2RL→21RL+2RL)(21)σ(E8)=15σ(∅¯→A+σ(1RD→∅)+12σ(A+1RD+2RD→21RD+2RD)(22)σ(E9)=15σ(∅¯→A)+σ(2RD→∅)+12σ(A+2RD+1RD→22RD+1RD).

As these are defined for each elemementary flux mode (EFM), they generally involve combinations of partial entropy productions associated to either a forward or reverse internal transformation and entropy exchange contributions coming from one or more of the input/output fluxes. Therefore, they can have have, in principle, any sign: either positive or negative. The relative weights for each partial entropy contribution are a consequence of the network stoichiometry. These weights simply count the number of times a given transformation occurs in the set of all the EFM pathways of the network. They must be included to avoid the multiple counting of any specific partial contribution. The partial entropy associated to each EFM pathway is obtained by dividing the partial entropy of the *i*th reaction (if it appears in that pathway) by the number of nonzero entries appearing in the *i*th row of the matrix E in Equation (8). Then, summing over the entropic contributions due to all nine extreme flux mode vectors yields the important identity [22]

(23) $$\sum_{i=1}^{9}\sigma \left(E_{i}\right)=\sigma -\sigma_{e}=\frac{dS}{dt}$$

establishing the intimate connection (first equality on the left) between the entropies of the extreme flux modes (pathways) and the balance equation (the second equality) [10] involving the entropy production, Equation (11), and the exchange entropy $\sigma_{e}$ [22]:

(24) $$\sigma_{e}=R\sum_{k}f\left(c_{k,in}-c_{k}\right)\ln\left(\frac{c_{k}}{c_{k,eq}}\right).$$

The reference concentrations $c_{k,eq}$ are determined from detailed balance and mass conservation: they correspond to isolating the reactor, having the chemical mass of the final NESS, from the open flow. In our case, they correspond to the species concentrations of the racemic state. The change in total system entropy is $\frac{dS}{dt}$. The first equality in (23) *resolves* the entropy balance between production and exchange in terms of the individual entropic contributions coming from each elementary pathway within the reaction network. At a NESS the entropy production is balanced by the entropy exchanged with the environment: $\sigma =\sigma_{e}\Leftrightarrow \frac{dS}{dt}=0$. Thus, the sum of the partial entropies over all EFMs vanishes identically at a NESS: $\sum_{i=1}^{9}\sigma \left(E_{i}\right)=0$, underscoring the importance of all the pathways, both productive and unproductive, open and closed, in contributing to this balance.

**Figure 4:**
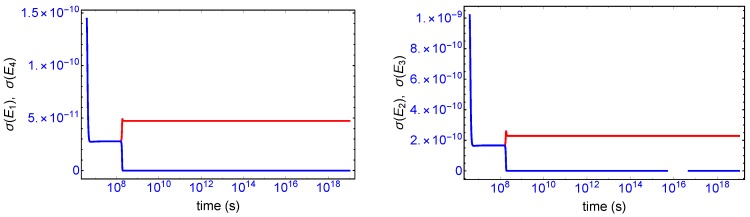
Bifurcation in the partial entropy productions $\sigma (E_{i})$, in units of $J\;K^{-1}s^{-1}L^{-1}$, associated with the enantiomeric pairs of extreme flux modes. **Left**: upper (red) branch $\sigma (E_{1})$, lower (blue) branch $\sigma (E_{4})$. **Right**: upper (red) branch $\sigma (E_{2})$, lower (blue) branch $\sigma (E_{3})$. See Table 1 and Equations (14)–(17).

**Figure 5:**
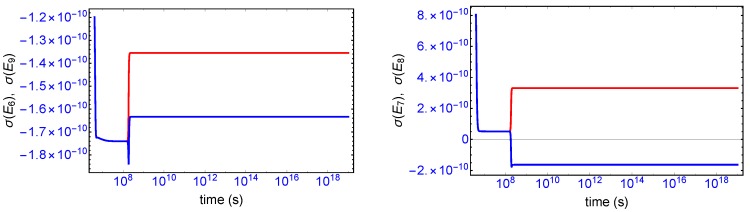
Bifurcation in the entropy productions $\sigma (E_{i})$, in units of $J\;K^{-1}s^{-1}L^{-1}$, associated with the enantiomeric pairs of extreme flux modes. **Left**: upper (red) branch $\sigma (E_{6})$, lower (blue) branch $\sigma (E_{9})$. **Right**: upper (red) branch $\sigma (E_{7})$, lower (blue) branch $\sigma (E_{8})$. See Table 1 and Equations (19)–(22).

**Figure 6:**
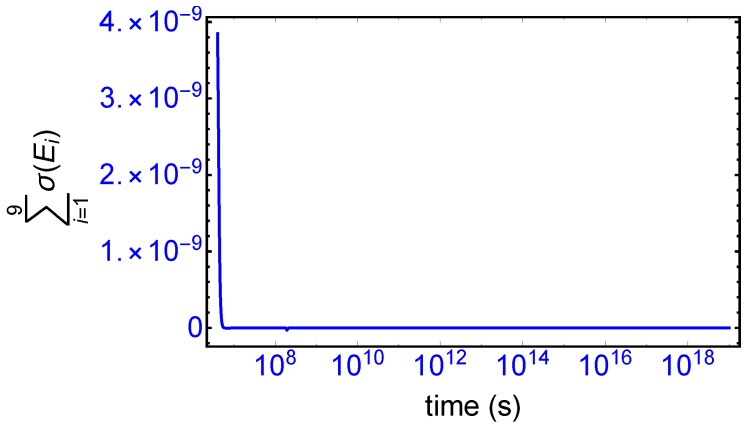
The sum of the partial entropies over all the extreme flux modes, in units of $J\;K^{-1}s^{-1}L^{-1}$. See Table 1 and Equations (14)–(22). Same parameters as employed in Figure 2. This sum vanishes at the final stable chiral non-equilibrium stationary state (NESS), as well as at the prior metastable racemic state, indicating that the entropy production exactly balances the exchange entropy $\sigma =\sigma_{e}$: see Equation (23). See text for behavior of the minuscule “spike” at $t\approx 2\times 10^{8}$ s.

**Figure 7:**
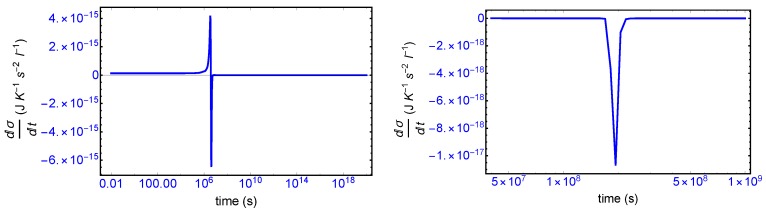
**Left**: the temporal derivative of the entropy production starts off positive, increasing in the vicinity of the production peak, and subsequently goes negative and then to zero during the metastable racemic phase and is also zero after SMSB as the system approaches the final stable chiral NESS. The small negative “spike” at approximately $2\times 10^{8}$ s (**right hand graph**) shows the derivative at the SMSB transition itself. Compare this derivative to the entropy production curve in Figure 3. We can resolve the total derivative into two independent contributions [10]: $\frac{d\sigma }{dt}=\frac{d_{F}\sigma }{dt}+\frac{d_{J}\sigma }{dt}$, Equation (26), to test validity of the GEC: see Figure 8.

Mirror symmetry breaking lifts the degeneracy in the values of the partial entropy productions associated with the EFM pair $\sigma \left(E_{1}\right),\sigma \left(E_{4}\right)$ and also with the pair $\sigma \left(E_{2}\right),\sigma \left(E_{3}\right)$, respectively, see Figure 4. From Table 1 the indicated EFM vectors represent enantiomeric pairs of internal closed reaction pathways; namely, the elementary pathways formed by the forward and reverse cross-catalyses for each enantiomer. Likewise, the pairs of partial entropies associated with the unidirectional open-flow pathways $\sigma \left(E_{6}\right),\sigma \left(E_{9}\right)$ and $\sigma \left(E_{7}\right),\sigma \left(E_{8}\right)$, respectively, also undergo bifurcations: see Figure 5. These latter open flow pathways involve contributions coming from the forward cross-catalyses together with the input/output flows, and their partial entropies can take on either positive or negative values. In all these initially mirror symmetric entropy productions, after SMSB the greater partial production is along the pathways involving the L-enantiomers (as we perturbed the initial racemic state with a slight excess of ${}^{2}R_{L}$). The sum of the entropy contributions over all the EFM vectors vanishes identically at any NESS: see Figure 6, confirming that production and exchange over all pathways are perfectly balanced both at the final chiral NESS, as well as at the prior meta-stable racemic state, as predicted by Equation (23). In networks with *M* EFMs, the sum is extended over *M*. We evaluate this sum to cover the time range subsequent to the large production peak in Figure 7. Note after this peak this sum is identically zero everywhere except at the SMSB transition itself, located approximately at $t\approx 2\times 10^{8}$ s, since the transition is a dynamic event. This tiny “spike” can be appreciated quite clearly examining a zoom of the temporal derivative of the entropy production itself: see the right hand graph in Figure 7.

## 5. Role of the Chemical Forces: The General Evolution Criterion

The entropy production Equation (11) can be written as a product over forces $F_{k}$ times flows $J_{k}$ [26,27]. For chemical reactions, the forces are proportional to the chemical affinities $A_{k}$, and the flows are differences in the corresponding reaction rates [10]:

(25) $$\sigma =\sum_{k}F_{k}J_{k}=\frac{1}{V}\sum_{k}\frac{A_{k}}{T}\frac{d\xi_{k}}{dt}\;\left\{\begin{array}{c} F_{k}=A_{k}/T=R\ln\left(\frac{v_{k,f}}{v_{k,r}}\right)\;\;\mathrm{forces}:\;\mathrm{chemical}\;\;\mathrm{affinities}\\ J_{k}=\frac{1}{V}\frac{d\xi_{k}}{dt}=\left(v_{k,f}-v_{k,r}\right)\;\;\mathrm{flows} \end{array}\right.$$

Now, the General Evolution Criterion (GEC) [26,27] states that the derivative of the entropy production with respect to the time-variation of the forces $d_{F}\sigma /dt=\sum_{k}\left(dF_{k}/dt\right)J_{k}$ obeys the inequality: $d_{F}\sigma /dt\leq 0$, (and vanishes =0 at the NESS). The total time derivative of σ receives two contributions which are generally independent in the nonlinear regime of non-equilibrium thermodynamics:

(26) $$\frac{d\sigma }{dt}=\underset{︸}{\frac{d_{F}\sigma }{dt}}+\frac{d_{J}\sigma }{dt}\;\;\;\;\mathrm{GEC}:\;\;\frac{d_{F}\sigma }{dt}\leq 0\;\;\left(=0\;\mathrm{at}\;\;\;\mathrm{NESS}\right)$$

Note: the derivative of the entropy production with respect to the time-variation of the flows *J*: $d_{J}\sigma /dt=\sum_{k}F_{k}\left(dJ_{k}/dt\right)$, by marked contrast, satisfies no such theorem. The proof of the GEC generally assumes time-independent boundary conditions and a variety of external physical forces can be taken into account [9,26,27], such as closed chemical systems and even certain types of open chemical systems [10]. We are interested in chemical reaction systems subject to input/output volumetric open-flow configurations, and thus seek the proper expression of the GEC valid for time-dependent open flow system architectures, not contemplated, to our knowledge, in the earlier demonstrations. A derivation of $d_{F}\sigma /dt$ for chemical reactions subject to time-dependent open flows is given in the Appendix A. An important point worth mentioning is that for open-flow systems, the chemical affinities (the forces) depend on *both* the internal reversible reactions and on the irreversible pseudoreactions, or matter fluxes, that maintain the system out of equilibrium. This explicit dependence is made manifest, see Equation (A18). We thus evaluate this expression for our model and confirm its validity and the compliance of the GEC over the full time range of our simulation.

We first consider the total derivative of the entropy production dσ/dt, displayed in Figure 7 (left hand panel). It starts off positive and becomes negative after the large entropy production peak, and remains negative (or zero) both before and after SMSB, indicating that stability of the final chiral NESS is achieved after the symmetry breaking. The momentary SMSB event itself can be appreciated clearly in the blow-up shown in the right-hand graph. The derivative is zero both during the prior metastable racemic interval as well as for the subsequent final chiral NESS. The small negative peak is the derivative of the racemic to chiral “step” in Figure 3. Thus the curves in Figure 7 represent faithfully the full time derivative of the entropy production Figure 3 over the entire time course of the evolution of this replicator system. Hence, since dσ/dt≤0 after SMSB, fluctuations about this chiral NESS decay in time, and the entropy production is minimized at this chiral NESS, and in the non-linear regime of non-equilibrium thermodynamics.

**Figure 8:**
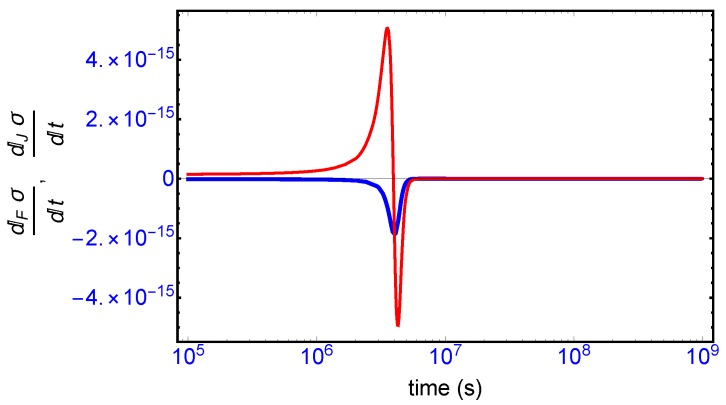
Blue curve: the change in the entropy production with respect to changes in the chemical forces F (the affinities), which is negative definite thoughout the entire time course and reaches zero at the final stable chiral NESS, and thus obeys the general evolution criterion (GEC) [9,10]; and Equation (A18). Red curve: the change in the entropy production with respect to changes in the flows J starts off positive then becomes negative after SMSB and then reaches zero from below on the approach to the final stable chiral NESS.

This complete time dependent behavior is resolved into two important contributions [10]: (i) one is due to the changes in the forces F (affinities) with respect to time (Figure 8, blue curve), and (ii) the other one is due to temporal changes in the flows or currents J (Figure 8, red curve). The general evolution criterion (GEC) [9,10] states that the changes in the forces (here, the chemical affinities) proceeds in such a way as to lower the value of the entropy production. There is however no general criterion (in the non-linear regime) yielding information about either the sign or the magnitude of the changes induced by the flows J. Here, we find the change in the flows J also becomes negative after the large production peak (Figure 8, red curve), thus also lowering the value of the entropy production. Both the force F and flow J contributions vanish on approach to the final chiral NESS. The GEC by itself is not a sufficient condition for SMSB: it does not imply dσ/dt≤0, which is the condition required for stability of a NESS. For the stability of any putative non-equilibrium stationary state (NESS), the changes in the flows J in the vicinity of the NESS must be bounded in absolute value by the changes in the forces F (by GEC these are negative definite): that is, $\frac{d_{J}\sigma }{dt}\leq \left|\frac{d_{F}\sigma }{dt}\right|$ in the neighborhood of a stationary state in order that dσ/dt→0. Such is the case here, and so the final chiral NESS is stable, and the entropy production is minimized.

We conclude from this analysis that the increase of the entropy production along the non-equilibrium racemic thermodynamic branch (see Figure 3) can lead to an instability in the case of SMSB. Chiral fluctuations can tip the system towards one of two degenerate non-racemic configurations, each one having a lower value of the entropy production relative to the previous unstable racemic branch.

## 6. Discussion

We have analyzed entropic pathway features underlying spontaneous mirror symmetry breaking for two chiral cross-catalyzed replicators. We have underscored the role of the open elementary flux modes (EFM), as defined by stoichiometric network analysis, for driving transitions from the unstable racemic thermodynamic branch to the branch of organized scalemic states, e.g., from racemic to homochiral, which takes place in the non-linear regime of non-equilibrium thermodynamics.

In our chiral replicator network, the EFMs can be organized into three distinguishable classes. Namely (i) internal closed pathways ($E_{1},E_{2},E_{3},E_{4}$) given by the four reversible pairs of cross-catalyses, (ii) open pathways ($E_{6},E_{7},E_{8},E_{9}$) composed by the ordered sequence: inflow of resource A, then the forward cross catalysis of an enantiomer, followed by the outflow of the enantiomer product of the prior forward cross-catalytic step, and (iii) the unreactive inflow/outflow of A through the reactor: $E_{5}$. SNA indicates that the open flux modes in (ii) are essential for entropy production, and the magnitude of this production is controlled by the nonnegative values of the respective convex parameters $j_{i}>0$, Equation (13), representing the matter flow through each respective EFM [18] and valid for any stationary state. The closed pathways (i) cannot and do not produce entropy by themselves. Finally, the nonreactive flow-through (iii) cannot produce entropy, in spite of being an open pathway traversing the reactor.

Numerical simulation of the differential rate equations associated with the scheme Equations (1)–(6) shows that the entropy production evolves to a minimum value, with respect to the prior metastable racemic state, and this minimization is coincident with the symmetry breaking transition. The EFMs shed light on how the entropy production and entropy exchanges are partitioned among all the pathways of the chemical network while maintaining the crucial balance at any NESS. The initial mirror symmetric degeneracy in the values of the enantiomeric flux pairs in the racemic configuration becomes lifted during SMSB. After SMSB, the partial entropy production is greater for the L-handed EFMs than for the R-handed EFMs. The sum of partial entropic contributions over nine EFMs goes to zero at the final chiral NESS, indicating the delicate balance Equation (23) between entropy production and exchange with the external environment, a fundamental thermodynamic condition for stationary open systems.

The GEC states that the generalized forces (here, the chemical affinities) evolve in such a way as to lower the entropy production. In open flow systems, the affinities depend on both the internal reversible reactions and on the irreversible matter fluxes. We derive this dependence Equation (A18) and calculate the GEC based on this analytic result, it agrees identically with the calculation of the GEC from the numerical simulation of the rate equations. This confirms the dependence of GEC on the matter fluxes and shows how the lowering of the entropy production predicted by GEC is achieved and consistent with the interdependence of the chemical reactions and the matter fluxes into and out from the reactor.

The EFM approach to entropy production, entropy transport and total entropy balance described here applies to any mass-action chemical reaction network in an open system and under a variety of system architectures. The usefulness of this SNA approach is the identification of the specific *unidirectional* matter fluxes responsible for the production of entropy and the role of external controlling factors, such as the input/outflow flows and their representation as pseudo-reactions. SNA leads to the expression for the rate of change of the total entropy (density) of the system dS/dt as the sum of the *partial* entropy productions over all the extreme flux modes (EFM), both the internal closed pathways and the open unidirectional ones. This yields insight into the coupling between chemical reactions and the input/output matter fluxes. At a NESS, the sum over all EFMs vanishes identically, and implies the balance between entropy production and entropy exchange. The entropy production is minimized at the final chiral NESS.

The hypercycle model involving solely the mutual cross catalyses was chosen for the purposes of illustrating in detail the methods and techniques. Naturally, more chemical realism can be built into the model by adding in the direct uncatalyzed synthesis of the replicators (e.g., from templates) [17]. This takes us from 9 to 25 EFMs. If we also add in the first-order enantioselective autocatalytic steps [17], and allow for two distinct achiral sources, then the number of EFMs will increase to 50. Including more replicators will also lead to more EFMs. Nevertheless, nothing qualitatively essential, from the non-equilibrium thermodynamic perspective, is gained from the increase in algebraic complexity and pathway diversity: the same basic qualitative features will emerge, similar in all important respects to those reported here.

## Figures and Tables

**Table 1:** Elementary flux modes $E_{i}$, the individual transformations they involve as enumerated in Equations (1)–(6), and their corresponding reaction subnetworks or pathways. The parity operation P, which acts on enantiomers in three dimensional space, induces symmetries on these vectors by relating pairs of enantiomeric extreme flux modes (EFMs) [21]. These pairs or *doublets* are $E_{1}\Leftrightarrow E_{4}$, $E_{2}\Leftrightarrow E_{3}$, $E_{6}\Leftrightarrow E_{9}$, and $E_{7}\Leftrightarrow E_{8}$. There is one *singlet*: $E_{5}$.

EFM:	Reactions	Subnetwork: Reaction Pathway
$E_{1}$	(10),(9)	$2\;\;{}^{2}R_{L}+{}^{1}R_{L}\to A+{}^{2}R_{L}+{}^{1}R_{L}$, $A+{}^{2}R_{L}+{}^{1}R_{L}\to 2\;\;{}^{2}R_{L}+{}^{1}R_{L}$
$E_{2}$	(8),(7)	$2\;\;{}^{1}R_{L}+{}^{2}R_{L}\to A+{}^{1}R_{L}+{}^{2}R_{L}$ $A+{}^{1}R_{L}+{}^{2}R_{L}\to 2\;\;{}^{1}R_{L}+{}^{2}R_{L}$
$E_{3}$	(2),(1)	$2\;\;{}^{1}R_{D}+{}^{2}R_{D}\to A+{}^{1}R_{D}+{}^{2}R_{D}$ $A+{}^{1}R_{D}+{}^{2}R_{D}\to 2\;\;{}^{1}R_{D}+{}^{2}R_{D}$
$E_{4}$	(4),(3)	$2\;\;{}^{2}R_{D}+{}^{1}R_{D}\to A+{}^{2}R_{D}+{}^{1}R_{D}$ $A+{}^{2}R_{D}+{}^{1}R_{D}\to 2\;\;{}^{2}R_{D}+{}^{1}R_{D}$
$E_{5}$	(14),(13)	A→∅, $\bar{\emptyset }\to A$
$E_{6}$	(13),(12),(9)	$\bar{\emptyset }\to A$ ${}^{2}R_{L}\to \emptyset$ $A+{}^{2}R_{L}+{}^{1}R_{L}\to 2\;\;{}^{2}R_{L}+{}^{1}R_{L}$
$E_{7}$	(13),(11),(7)	$\bar{\emptyset }\to A$ ${}^{1}R_{L}\to \emptyset$ $A+{}^{1}R_{L}+{}^{2}R_{L}\to 2\;\;{}^{1}R_{L}+{}^{2}R_{L}$
$E_{8}$	(13),(5),(1)	$\bar{\emptyset }\to A$ ${}^{1}R_{D}\to \emptyset$ $A+{}^{1}R_{D}+{}^{2}R_{D}\to 2\;\;{}^{1}R_{D}+{}^{2}R_{D}$
$E_{9}$	(13),(6),(3)	$\bar{\emptyset }\to A$ ${}^{2}R_{D}\to \emptyset$ $A+{}^{2}R_{D}+{}^{1}R_{D}\to 2\;\;{}^{2}R_{D}+{}^{1}R_{D}$

**Table 2:** The partial entropy productions per unit volume σ for chemical transformations of the form A+B+C⇌2B+C and for the irreversible pseudo-reactions →X and Y→, see [22]. The reference equilibrium concentrations ${\left[X\right]}_{eq},{\left[Y\right]}_{eq}$, are those established when the reactor, and with the chemical mass of the final non-equilibrium stationary state (NESS), is isolated from the open flow; see text.

Transformation	Partial Entropy Production/Exchange
A+B+C→2B+C	$\sigma \left(A+B+C\to 2B+C\right)=R\;k_{+}\left[A\right]\left[B\right]\left[C\right]\ln\left(\frac{k_{+}\left[A\right]}{k_{-}\left[B\right]}\right)$
2B+C→A+B+C	$\sigma \left(2B+C\to A+B+C\right)=R\;k_{-}{\left[B\right]}^{2}\left[C\right]\ln\left(\frac{k_{-}\left[B\right]}{k_{+}\left[A\right]}\right)$
→X	$\sigma \left(\to X\right)=R\;f{\left[X\right]}_{in}\ln\left(\frac{{\left[X\right]}_{eq}}{\left[X\right]}\right)$
Y→	$\sigma \left(Y\to \right)=R\;f\left[Y\right]\ln\left(\frac{\left[Y\right]}{{\left[Y\right]}_{eq}}\right)$

## Abbreviations

The following abbreviations are used in this manuscript:

SMSB	Spontaneous mirror symmetry breaking
NESS	Non-equilibrium stationary state
SNA	Stoichiometric network analysis
EFM	Extreme flux mode
GEC	General evolution criterion
CSTR	Continuously stirred tank reactor

## Appendix A. GEC for Chemical Reactions in Open-Flow

Consider *r* pairs of reversible transformations (w=1,2,…,r) obeying mass action kinetics and for *n* species:

(A1) $$\sum_{u=1}^{n}\beta_{uw}^{+}X_{u}\overset{k_{w}^{+}}{\underset{k_{w}^{-}}{⇌}}\sum_{u=1}^{n}\beta_{uw}^{-}X_{u},$$

subject to the irreversible “pseudo-reactions” (volumetric open-flow terms) for u=1,…,n:

(A2) $$\begin{array}{ccc} & \overset{k_{in,u}{\left[X_{u}\right]}_{in}}{\to } & X_{u},\;\left(\mathrm{inflow}\right) \end{array}$$

(A3) $$\begin{array}{ccc} X_{u} & \overset{k_{out,u}}{\to } & \;(\mathrm{outflow}). \end{array}$$

The *effective* reaction rate constants are $k_{in,u}=q/V=k_{out,u}$, where q=l/s is the volume *l* of fluid entering/exiting the reactor of volume *V* per second *s*, and ${\left[X_{u}\right]}_{in}$ is the fixed concentration of species *u* flowing into the reactor (across the system boundary). These flows maintain the system volume V constant. Inflows and outflows need not exist in matched pairs: that is, a given species can flow out of the reactor (say as a product) without necessarily flowing into it, etc. A species flowing in at a fixed input concentration can however flow out of the reactor with an instantaneous concentration that will generally be distinct from its fixed input value.

The forward and reverse reaction rates for the pairs of reversible reactions in Equation (A1) are:

(A4) $$r_{w}^{\pm }=k_{w}^{\pm }\prod_{u=1}^{n}{\left(x_{u}\right)}^{\beta_{uw}^{\pm }},\;x_{u}=\left[X_{u}\right].$$

In the following $\nu_{uw}$ denote the elements of the n×r stoichiometry matrix corresponding to the *reversible* reactions only (A larger stoichiometric matrix encompassing the reversible reactions and the pseudoreactions, by regarding all the one-way transformations as irreversible, is also possible, see Section 2 and [21]):

(A5) $$\nu_{uw}=\left(\beta_{uw}^{-}-\beta_{uw}^{+}\right).$$

The changes in the concentrations depend on the open-flows and reversible reactions as follows (ξ is the extent of reaction):

(A6) $$V\;dx_{u}=\left[d\phi_{u}^{e,in}-d\phi_{u}^{e,out}\right]+\sum_{w}\nu_{uw}\;d\xi_{w},$$

The rate equations for the concentrations can be split into two contributions, $\frac{d_{e}X_{u}}{dt}$ are the changes in the concentration due to transport processes (volumetric flows, pseudoreactions) and a part due to reversible chemical reactions: $\frac{d_{i}X_{u}}{dt}$. The latter can be written in terms of the stoichiometric matrix for the *r*-reversible reactions, and the open flow terms, represented by the pseudo-reactions, as follows:

(A7)dxudt=dexudt+dixudt,(A8)dxudt=[dϕue,indt−dϕue,outdt]/V+∑w=1rνuwdξwVdt,(A9)dxudt=kin,u[Xu]in−kout,uxu+∑w=1rνuwvw.

For *steady states* (ss) we have $\frac{dx_{u}}{dt}=0$ so that

(A10) $$\begin{array}{ccc} \left(k_{in,u}{\left[X_{u}\right]}_{in}-k_{out,u}\;{\left[X_{u}\right]}_{ss}\right) & = & -\sum_{w=1}^{r}\nu_{uw}{\bar{v}}_{w}. \end{array}$$

For open flow, the affinities are functions of both the extents of reaction ξ and the input/output fluxes:

(A11)Ak=Ak({ξ},{ϕe}),(A12)⇒∂Ak∂t=∑j=1r∂Ak∂ξjdξjdt+∑i=1n∂Ak∂ϕiedϕiedt,(A13)=∑j=1r∂Ak∂ξjdξjdt+∑i=1n∂Ak∂ϕie,indϕie,indt+∑i=1n∂Ak∂ϕie,outdϕie,outdt.

where

(A14) $$\begin{array}{ccc} \frac{\partial A_{k}}{\partial \phi_{i}^{e,in}} & = & -RT\frac{\nu_{ik}}{x_{i}}, \end{array}$$

(A15) $$\begin{array}{ccc} \frac{\partial A_{k}}{\partial \phi_{i}^{e,out}} & = & +RT\frac{\nu_{ik}}{x_{i}}, \end{array}$$

hence, when substituted into (A13), leads to

(A16) $$\begin{array}{ccc} \frac{\partial A_{k}}{\partial t} & = & \sum_{j=1}^{r}\frac{\partial A_{k}}{\partial \xi_{j}}\frac{d\xi_{j}}{dt}-RT\sum_{i=1}^{n}\frac{\nu_{ik}}{x_{i}}\left[\frac{d\phi_{i}^{e,in}}{dt}-\frac{d\phi_{i}^{e,out}}{dt}\right]. \end{array}$$

At a NESS, we prove the temporal derivative of the affinities vanishes ($h_{u}=1/{\left[X_{u}\right]}_{ss}$):

(A17) $$\begin{array}{ccc} \frac{\partial A_{k}}{\partial t}{|}_{NESS} & = & \sum_{j=1}^{r}\left(-RT\sum_{u=1}^{n}\nu_{uk}\nu_{uj}h_{u}\right)\frac{d\xi_{j}}{dt}-RT\sum_{i=1}^{n}\nu_{ik}h_{i}\left[-\sum_{w=1}^{r}\nu_{iw}\frac{d\xi_{w}}{dt}\right]\\ & = & 0, \end{array}$$

the partial derivatives of the affinities with respect to the extents is given in [22].

Finally, the derivative of the entropy production with respect to changes in the forces (chemical affinities) in the presence of open-flow is given by (set V=1):

(A18) $$\frac{d_{F}\sigma }{dt}=\sum_{k=1}^{r}\left(\sum_{j=1}^{r}\frac{1}{T}\frac{\partial A_{k}}{\partial \xi_{j}}\frac{d\xi_{j}}{dt}-R\sum_{i=1}^{n}\frac{\nu_{ik}}{x_{i}}\left[\frac{d\phi_{i}^{e,in}}{dt}-\frac{d\phi_{i}^{e,out}}{dt}\right]\right)\frac{d\xi_{k}}{dt}\leq 0,$$

and is the extension of the GEC [9,10,26,27] to chemical reactions in open-flow.
